# Urogynecology in obstetrics: impact of pregnancy and delivery on pelvic floor disorders, a prospective longitudinal observational pilot study

**DOI:** 10.1007/s00404-021-06022-w

**Published:** 2021-03-22

**Authors:** Russalina Stroeder, Julia Radosa, Lea Clemens, Christoph Gerlinger, Gilda Schmidt, Panagiotis Sklavounos, Zoltan Takacs, Gabriele Meyberg-Solomayer, Erich-Franz Solomayer, Amr Hamza

**Affiliations:** 1 Department of Gynecology, Obstetrics and Reproductive Medicine, University Medical School of Saarland, Kirrberger Straße, 66421 Homburg, Saar Germany; 2 Department of Obstetrics and Prenatal Medicine, Kantonspital Baden, Im Ergel 1, 5400 Baden, Switzerland

**Keywords:** Pelvic floor disorders, POP-Q, Translabial ultrasound, Quality of life, Preventive treatment strategies

## Abstract

**Purpose:**

To assess changes in the pelvic floor anatomy that cause pelvic floor disorders (PFDs) in primigravidae during and after pregnancy and to evaluate their impact on women’s quality of life (QoL).

**Methods:**

POP-Q and translabial ultrasound examination was performed in the third trimester and 3 months after delivery in a cohort of primigravidae with singleton pregnancy delivering in a tertiary center. Results were analyzed regarding mode of delivery and other pre- and peripartal factors. Two individualized detailed questionnaires were distributed at 3 months and at 12 months after childbirth to determinate QoL.

**Results:**

We recruited 45 women, of whom 17 delivered vaginally (VD), 11 received a vacuum extraction delivery (VE) and 17 a Cesarean section in labor (CS). When comparing third-trimester sonography to 3 months after delivery, bladder neck mobility increased significantly in each delivery group and hiatal area increased significantly in the VD group. A LAM avulsion was found in two women after VE. Connective tissue weakness (*p* = 0.0483) and fetal weight at birth (*p* = 0.0384) were identified as significant risk factors for the occurrence of PFDs in a multivariant regression analysis. Urinary incontinence was most common with 15% and 11% of cases at 3, respectively, 12 months after delivery. 42% of women reported discomfort during sexual intercourse, 3 months after delivery and 24% 12 months postpartum. Although 93% of women engage a midwife after delivery, only 56% participated in pelvic floor muscle training.

**Conclusion:**

Connective tissue weakness and high fetal weight at birth are important risk factors for the occurrence of PFDs. Nevertheless, more parturients should participate in postpartal care services to prevent future PFDs.

**Supplementary Information:**

The online version contains supplementary material available at 10.1007/s00404-021-06022-w.

## Introduction

Pelvic floor disorders (PFDs), including urinary incontinence (UI), anal incontinence (AI) and pelvic organ prolaps (POP), affect up to 30% of the adult female population [1–3]. The prevalence is likely to grow in the aging population, consequently causing an increase in health-care expenditures [4, 5]. Knowledge of the mechanisms leading to PFDs in later life offers the opportunity to initiate preventive treatment strategies. The etiology of PFDs is yet complex and multifactorial. Genetic predisposition, lifestyle factors, BMI, aging, pregnancy and delivery all play an important role [1, 3, 6–17]. During gestation, the weight gain and increase in uterine weight increases the intra-abdominal pressure. Therefore, ligamentous structures and pelvic floor muscles are overstretched [6]. The expanding retrovesical angle increases furthermore the BNM and results in UI [18]. Hormonal and mechanical changes during pregnancy also contribute to impairment of normal pelvic floor function [19]. This explains why Cesarean section (CS) is not entirely protective against development of PFDs [20]. Childbirth can furthermore result in myogenic, neurogenic damage and/or connective tissue damage. In most cases, these changes are reversible and the pelvic floor muscle function recovers during the first year after delivery [21, 22]. Pelvic floor changes appear to be irreversible in only 5–20% and then leading to PFDs in later life [20]. This collective may benefit from preventive treatment strategies (PTS), such as pelvic floor muscle training (PFMT), physiotherapy, pessar therapy, use of topical estrogen therapy, electrostimulation, etc. [23, 24] PFDs give rise to sexual disorders, which results in detrimental effects on women's quality of life (QoL) [25]. However, the physiopathology of PFDs is not fully established and longitudinal studies with objective quantitative data are rare [20].

Changes in pelvic floor anatomy such as: increased bladder neck mobility (BNM), stretching of the levator hiatus and injuries of the levator ani muscle (LAM avulsion) were already detected in previous studies [26–35]. The purpose of this study was to evaluate changes in pelvic floor biometry and function and its clinical implications in and after pregnancy using POP-Q, translabial ultrasound and questionnaires.

This should help to identify former primigravidae with high risk factors for occurrence of PFDs and help inform them about possible PTS.

## Methods

The recruitment took place from May to October 2017. Our prospective longitudinal observational study included only primigravidae with uncomplicated singleton pregnancy planned for delivery in our tertiary center, who agreed to participate after signing the written informed consent. Exclusion criteria were only age < 18 years, multigravidae and multiples.

**Fig. 1:**
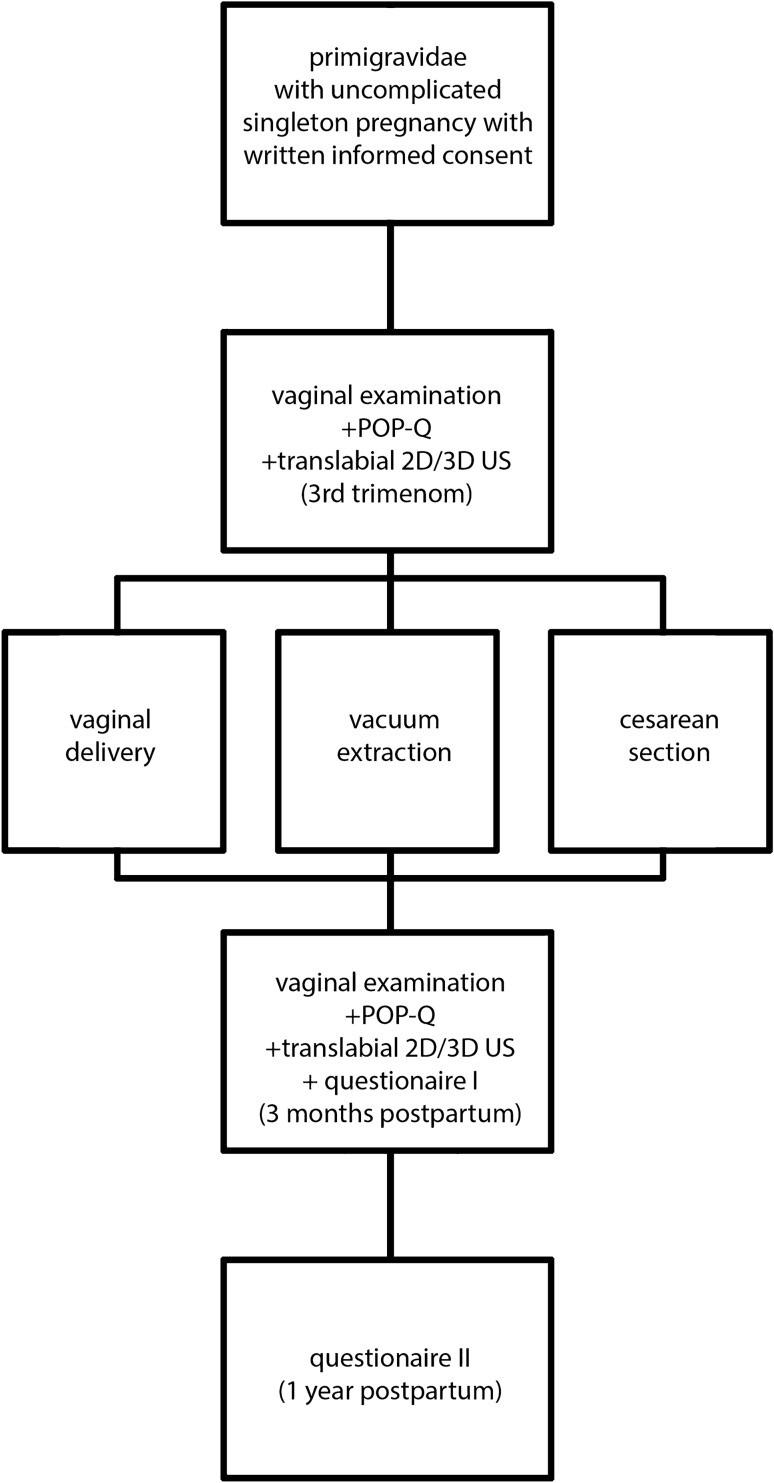
Overview of study procedure

45 out of 56 returned for follow-up 3 months after delivery. The study protocol was approved by the local Ethical Committee. On recruitment, we performed a vaginal examination, a POP-Q test and translabial 2D/3D ultrasound examination in the third trimester of pregnancy (34th–36th gestational week) and 3 months after delivery, as illustrated in Fig. 1.

Primary outcome parameters were anatomic changes of the pelvic floor as measured by ultrasound and clinical examination (bladder neck mobility, changes of the levator hiatus, occurrence of pelvic floor prolaps). Secondary outcomes were possible limitations in quality of life, occurrence of incontinence symptoms, physical activity, participation in pelvic floor muscle training and changes in sexual function.

Vaginal examination was performed in the lithotomy position. Genital descent was evaluated at rest and on Valsalva maneuver (VM) using the POP-Q grading system proposed by the International Continence Society [36].

Translabial ultrasound examination was performed by one of two investigators (A.H. and R.S.) who had ultrasound experience for several years. A GE Voluson 730 ultrasound system (GE Healthcare, Hoevelaken, The Netherlands) with RAB 4—8 MHz curved array 3D/4D ultrasound transducer was used. The ultrasound transducer was covered sterile and placed vertically on the perineum in the sagittal plane with the participant in a supine position after micturition. Datasets were obtained at rest and on maximum VM.

The pelvic floor was initially visualized in B-mode to evaluate the BNM, which is strongly associated with the onset of UI [32, 37, 38]. To visualize the BNM, we measured the bladder neck descent and the retrovesical angle (ß) at rest and on VM. The bladder neck descent was measured relatively to the inferior margin of the symphysis pubis as an immobile reference point as previously described by Dietz HP [39, 40]. The retrovesical angle (ß) was measured between the dorsal urethral axis and the axis of the floor of the bladder, as previously described by Green et al. [41]. The normal angle was defined as 90–100° in non-pregnant women. It increases in women with UI [38, 42].

The levator hiatal dimensions were measured in the axial plane of least levator hiatal gap by 3D ultrasound, identified in the mid-sagittal image as the minimal distance between the posterior-inferior margin of the symphysis pubis and anorectal junction. We obtained the hiatal measurements using the rendering technique as described previously by Dietz [34, 43]. The following measurements were collected at rest and on VM to estimate the contractility and distensibility of the levator hiatus: (1) anteroposterior diameter of the levator hiatus (LH-ap), (2) the levator hiatal transverse diameter (LH-rl) and (3) the area of the levator hiatus (LH-area), bordered by the pubovisceral muscle, symphysis pubis and the inferior pubic ramus. Ballooning of the levator hiatus was defined as a distension of the hiatal area > 25 cm^2^ which is associated with POP [44–46].

We also assessed LAM integrity. LAM avulsion was diagnosed if the distance between the center of the urethra and the LAM insertion (levator-urethra gap) was > / = 25 mm. LAM avulsion is also associated with POP [44, 45, 47]. Offline analyses of the rendered volume datasets were performed.

Two individualized non-validated detailed questionnaires (appended in the attachment) were distributed at 3 months postpartum visit and 12 months after childbirth on postal way to assess QoL, occurrence of PFDs and women’s behavior after childbirth (weight, breast feeding, postpartal engagement of a midwife, participation at pelvic floor muscle training or other specialized treatments, activity and sports).

The following clinical data and intrapartal parameters were received from the medical records database: (1) gestational age at childbirth, (2) delivery mode, (3) anesthesia, (4) induction of labor (IOL), (5) medication during labor, (6) length of the delivery periods, (7) fetal measurements, (8) perineal and other genital injuries.

Data base was edited in Microsoft Excel Mac 2011 (Redmond, USA) and anonymized before starting statistical assessment. Following the intention-to-treat principle all patients with available data were included in the analyses. Categorical variables were described using absolute and relative frequency counts whereas continuous variables were described using the number of observations, mean, and standard deviation. As a result of the low frequencies of UI, AI, and mixed incontinence the dichotomized variable incontinence (yes/no) was used as the dependent variable in the logistic regressions. A stepwise selection procedure with limits 0.1 for inclusion and/or exclusion of variables was used. Owing to the explorative nature of the study, a comparison-wise two-sided significance level of 5% was applied. The statistical analyses were carried out using SAS version 9.4 software (SAS Inc. Cary, NC, USA).

## Results

We recruited 56 women, of whom 45 (80.4%) completed the study. 11 women were excluded from the study due to refusal of further participation (*n* = 8), delivery in another hospital (*n* = 2) and intrauterine fetal death (*n* = 1). Patients’ characteristics are displayed in Table 1. Table 2 shows peripartal parameters and fetal characteristics. The most frequent indication for induction of labor was post-term pregnancy. The most frequent indication for CS and VE was pathological fetal monitoring during first, respectively, second-stage of labor.

**Table 1:** Patient characteristics

Characteristics	
Age [years]	31 (range 18—40)
Body mass index before pregnancy [kg/m^2^]	25 ± 5.1
Weight increase through pregnancy [kg]	15 ± 6.6
Connective tissue weakness	23 (51.1%)
Gestational diabetes	6 (13%)

**Table 2:** Peripartal parameters and fetal characteristics

Peripartal parameters	Number of participants
Gestational age at delivery [weeks]	40 ± 1.6
Induction of labor	18 (40%)
Mode of delivery	
Vaginal delivery	17 (37.8%)
Vacuum assisted delivery	11 (24.4%)
Cesarean section in labor	17 (37.8%)
Peridural anesthesia	12 (27%)
Length of delivery period [min]	
First stage of labor	208
Second stage of labor	66
Total	274
Episiotomy	18 (40%)
III° perineal tear	4 (9%)
Fetal weight [g]	3213 (range 2100–4200)
Fetal head circumference [cm]	34 (range 31–37.5)

Differences in pelvic floor function before and after childbirth depending on different delivery mode are presented in Table 3. Comparing third-trimester sonography to 3 months postpartum, BNM increased significantly on VM from 2 to 10 mm in each delivery group. The retrovesical angle decreased from 132.6 ± 9.74° to 128.3 ± 15.6°, which was not significant and the hiatal area increased on VM from mean 13.37–14.53 cm^2^. The increase of the hiatal area was only significant in the VD group in the paired t test. Ballooning of the hiatal area was found in two women after VD. A LAM avulsion was diagnosed in two women 3 months after VE delivery.

**Table 3:** Quantitative parameters assessed in translabial sonography during pregnancy and after different modes of delivery

	During pregnancy (34-36th week) *n* = 45	Spontaneous delivery *n* = 17		Vacuum extraction *n* = 11		Cesarean section *n* = 17	
	mean ± SD	mean ± SD	*p* value	mean ± SD	*p* value	mean ± SD	*p* value
BNM [cm]	0.2	1.04 ± 0.26	< 0.0001*	0.86 ± 0.32	.0072*	1.08 ± 0.33	< 0.0001*
Retrovesical angle [°]	132.6 ± 9.74	132.08 ± 14.53	0.76	133.64 ± 7.45	.41	122.06 ± 18.55	0.51
Hiatal area on VM [cm^2^]	13.37	16.81 ± 4.67	0.0117*	13.35 ± 1.97	.49	13.55 ± 2.86	0.66
LAM avulsion	0	0		2		0	
Ballooning	0	2		0		0	

Hiatal area was significantly smaller after VE (*p* value = 0.04) and CS (*p* value = 0.03) than after spontaneous delivery

*BNM* bladder neck mobility, *LAM* levator ani muscle

*p* value (paired *t* tests comparing values during pregnancy and post-partum, significant *p* value (< 0.05) marked with an asterisk)

POP-Q test showed an anterior stage 1 in four women in the third trimester and in seven cases 3 months after delivery. We diagnosed three cases of posterior POP-Q stage 1 in the third trimester and five cases 3 months after delivery.

Among pre- and peripartal parameters connective tissue weakness as existence of cellulitis or striae (*p* = 0.0483) and fetal weight at birth (*p* = 0.0384) were identified as significant risk factors for the occurrence of PFDs in a multivariant regression analysis.

12 (26.7%) women reported UI during pregnancy. UI was reported 3 months and 12 months postpartum in seven and five, AI in three and two and a combined urinary and anal incontinence in seven and one woman, respectively. Seven out of 11 (64%) participants, who underwent VE showed symptoms 3 months postpartum, although there was no significant association between mode of delivery and the occurrence of PFDs.

19 (42%) and 11 (24%) women reported discomfort during sexual intercourse 3 and 12 months after delivery, respectively. Dyspareunia was stated by 12 (27%) and 7 (16%) cases 3 and 12 months postpartum, respectively. 17 (38%) stated no changes during intercourse while 9 participants (20%) had no intercourse throughout the survey. In still 11 (24%) of the participants symptoms of discomfort during sexual intercourse remained 12 months postpartum.

Although 93% of women are followed up by a midwife after delivery, only 56% participated in PFMT and 35% of women with incontinence symptoms used additional specialized treatments as physiotherapy or electrostimulation.

## Discussion

To our knowledge, we are one of few studies evaluating the occurrence of PFDs in a prospective longitudinal setting (during pregnancy and after childbirth) using a combination of pelvic floor examination, POP-Q and translabial 2D and 3D ultrasound. Furthermore, we investigate the impact of changes in pelvic floor on women’s QoL with the help of individualized questionnaires. This study may offer a better understanding of the pelvic floor function in context of gestation and delivery and its clinical relevance.

Most studies applied only one method for evaluating pelvic organs changes, e.g. POP-Q [22, 48] or perineal ultrasound [30, 49] with or without clinical correlation through a questionnaire. To our knowledge, only Jundt et al. [27] used a similar setup combining a questionnaire with POP-Q and perineal ultrasound in primiparous women between 32 and 37 weeks’ and 6 months postpartum.

Only few prospective studies with longitudinal follow-up using questionnaires provide information on all aspects of PFDs and/or include antenatal assessment. Macarthur et al. [50] e.g., only evaluated AI in context with childbirth and Van Brummen et al. [51] focused on UI after delivery.

Our study population consists of women with an advanced age of first gestation, a high BMI and a high percentage of instrumental deliveries (24%) or CS (38%), which is representative in our university hospital. Knowing this, the findings of this study cannot be generalized due to possible demographic differences.

The observational period chosen in our study extended from the third trimester to one year after delivery. Clinical measurements and a questionnaire were applied in third trimester and 3 months postpartum and only a questionnaire after 1 year with the aim to avoid poor follow-up rates. Our drop-out rate was 19.6% compared to Elenskaia et al. [48] e.g., who performed a similar study with a drop-out rate of 46.7%.

Our first exam was performed in third trimester. Therefore our results did not include early pregnancy patients as presented by Dietz et al. [30], who performed a prospective observational study in nulliparous women starting at 6–18 pregnancy weeks.

To avoid using several different validated questionnaires to cover the questions of interest, we decided to use two individualized none-validated questionnaires in contrast to other authors [48]. In addition, Zuchelo et al. [52] postulated that validated questionnaires are developed for women with incontinence and are not suitable for the postpartum period. They recommended new designed questionnaires to improve early and specific approach for this period of life. We used a self-administered questionnaire to avoid physician dependent bias.

Comparing third trimester 2D sonography to 3 months postpartum, BNM increased significantly on VM in all delivery groups. This was idem to the findings of Peschers et al. [53] who compared primiparae to nulligravid volunteers. They reported an increase in BNM in most women delivering vaginally. Increased BNM is associated with occurrence of UI [27, 49]. UI was most frequently reported among the PFDs with 31% of cases, 42.9% of whom already occurring during pregnancy.

In the VD group the hiatal area increased significantly on VM and 4% of women even present a ballooning in the 3D sonography. 4% of women had a LAM avulsion after VE. Other study groups report higher incidences. Shek et al. [54] e.g., detected 13% LAM avulsion and 28.5% ballooning in 367 primiparous women undergoing perineal ultrasound 2, respectively. 6 months postpartum.

The prevalence of POP was low in our collective with no cases of high-grade pelvic organ changes during pregnancy and after delivery. A prospective Norwegian study [22] of 300 nulliparous women also reported a low prevalence for POP during pregnancy from 0 to 10% and a recovery to baseline of all POP-Q points except the cervix at 12 months postpartum.

Connective tissue weakness and fetal weight at birth were identified as significant risk factors for PFDs in a multivariant regression analysis. This is comparable to the findings from other study groups. Miedel et al. [55] also reported about markers suggestive for congenital susceptibility (family history and conditions suggestive for weakened connective tissue) and non-obstetrical strain on the pelvic floor (overweight/obesity, heavy lifting and constipation). This implies an individual predisposition towards PFDs. Keane et al. compared premenopausal nulliparous women with urodynamically proven genuine stress incontinence with healthy controls and showed a defect in their connective tissue responsible for the UI [56]. In accordance to our results, Gyhagen et al. [16] found a significant correlation between high birth weight, LAM avulsion and genital descensus.

Conventionally known risk factors [16, 57], e.g., maternal age, obesity, breastfeeding, length of the second delivery period, fetal head circumference, perineal rupture were not found significant in our study due to small number of cases.

Our results suggest that VE may be associated with more UI and/or AI occurrence, since 64% of women were symptomatic after VE. Jundt et al. [27] already showed a relation between increase of BNM and VE.

From the questionnaires we concluded, that 27% of women stated dyspareunia 3 months postpartum and 16% 1 year after childbirth. This is comparable to the findings of Connolly et al. [58] reporting dyspareunia in 17% after childbirth.

There were few findings of concern in the questionnaires. Although 93% of women engage a midwife after delivery, only 56% participated in PFMT. Only 35% of women with incontinence symptoms used additional specialized treatments. Although several randomized controlled trials [23, 24] have clearly shown that intensive and supervised PFMT with/without biofeedback is effective for providing PFDs. A possible explanation might be the lack of time due to motherhood duties or the limited symptoms resulting from pelvic floor changes at this time. Informing women properly about PFD development and options of PTS, appears to be necessary to prevent PFDs in later life time and reduce the costs of managing future cases of incontinence.

The major limitation in our study was the number of cases, which was too low to establish statistically valid outcomes. We, therefore, classify our study as a pilot study. Further investigations are needed to verify our findings. We plan to do so in a multicenter fashion.

## Conclusion

Both, pregnancy and delivery have an effect on changes of the female pelvic floor. BNM increases after delivery when compared to pregnancy. Connective tissue weakness and high fetal weight at birth are identified as significant risk factors for the occurrence of PFDs in our study population. Although most of the parturient engage a postpartal midwifery care, yet only half of the patients participate in PFMT and only one third of women with incontinence symptoms used indicated specialized treatments. The participation rate in postpartal care services should be improved to implement an effective prevention for future PFDs.

## Supplementary Information

Below is the link to the electronic supplementary material.

Supplementary file1 (DOCX 20 KB)
