# Supplementary material for: Urogynecology in obstetrics: impact of pregnancy and delivery on pelvic floor disorders, a prospective longitudinal observational pilot study
Source: Arch Gynecol Obstet. 2021 Mar 22;304(2):401–8. doi: 10.1007/s00404-021-06022-w (PMC8277616; doi:10.1007/s00404-021-06022-w)
Supplement: Supplementary file 1 — Supplementary file1 (DOCX 20 KB) [file 404_2021_6022_MOESM1_ESM.docx]

**ATTACHMENT**

1. Questionnaire I
2. Questionnaire II

(1)

| Name:  Given name: |  |
| --- | --- |
| Date of Birth:  Age: |  |
| Height (cm):  Current weight (kg):  Weight before pregnancy (kg):  Increase of weight throughout pregnancy (kg): |  |
| How many pregnancies have you had before? |  |
| Date of delivery:  Mode of delivery:  (1) Spontaneous delivery  (2) Vacuum delivery  (3) Cesarean section |  |
| Fetal weight (g):  Fetal head circumference (cm): |  |
| Did you suffer from urinary incontinence **before** pregnancy?  If your answer is yes, please specify when and in which situation (for example: during laughing, during coughing, during activity or sports etc.) | Yes: 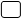  When and in which situation:  No: 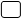 |
| Did you suffer from urinary incontinence **during** pregnancy?  If your answer is yes, please specify when and in which situation (for example: during laughing, during coughing, during activity or sports etc.) | Yes: 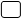  When and in which situation:  No: 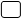 |
| Did you suffer from urinary incontinence **after** pregnancy?  If your answer is yes, please specify when and in which situation (for example: during laughing, during coughing, during activity or sports etc.) | Yes: 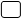  When and in which situation:  No: 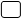 |
| Do you **still** suffer from urinary incontinence, 3 months after delivery? | Yes: 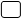  No: 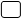 |
| Did you suffer from anal incontinence **before** pregnancy?  If your answer is yes, please specify  for example in form of involuntary expulsion of gas, liquid or solids from the lower bowel. | Yes: 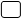  Specify:  No: 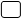 |
| Did you suffer from anal incontinence **during** pregnancy?  If your answer is yes, please specify. | Yes: 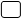  Specify:  No: 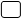 |
| Did you suffer from anal incontinence **after** pregnancy?  If your answer is yes, please specify. | Yes: 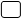  Specify:  No: 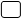 |
| Do you **still** suffer from anal incontinence, 3 months after delivery? | Yes: 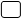  No: 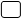 |
| Do you feel ashamed speaking about incontinence in context of pregnancy and delivery with others for example your midwife/ gynecologist/ friends? | Yes: 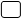  No: 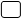 |
| If you do not feel ashamed, to whom do you speak about these problems? |  |
| Do you currently participate or have you participated in pelvic floor muscle training (PFMT) after delivery? | Yes: 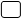  No: 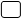 |
| Were you followed up by a midwife after delivery? | Yes: 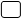  No: 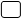 |
| Did you suffer from connective tissue weakness for example cellulite, varicose veins or striae? | Yes: 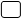  No: 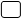 |
| Do you practice sports?  If your answer is yes, how often did you practice per week? | Yes: 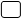  How often:  No: 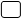 |
| Have you already practiced sexual intercourse after delivery?  If your answer is yes, how many weeks after delivery did you practice sexual intercourse? | Yes: 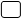  When:  No: 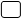 |
| Did you feel any changes during sexual intercourse after delivery?  If your answer is yes, please specify. | Yes: 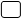  Specify:  No: 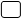 |
| Did you feel pain during sexual intercourse after delivery? | Yes: 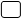  No: 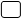 |
| Did you register foreign body feeling during sexual intercourse after delivery? | Yes: 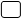  No: 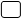 |

(2)

| Name:  Given name: |  |
| --- | --- |
| Date of Birth:  Age: |  |
| Height (cm):  Current Weight (kg): |  |
| Are you pregnant again?  If your answer is yes, please tell us your pregnancy week. | Yes: 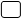  Pregnancy week:  No: 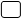 |
| Do you suffer from urinary incontinence, 1 year after delivery?  If your answer is yes, please specify when and in which situation (for example: during laughing, during coughing, during activity or sports etc.) | Yes: 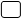  When and in which situation:  No: 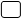 |
| Do you **still** suffer from anal incontinence, 1 year after delivery?  If your answer is yes, please specify  for example in form of involuntary expulsion of gas, liquid, or solids from the lower bowel. | Yes: 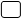  Specify:  No: 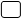 |
| Do you feel ashamed speaking about incontinence in context of pregnancy and delivery with others for example your midwife/ gynecologist/ friends? | Yes: 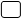  No: 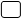 |
| If you do not feel ashamed, who do you talk about these problems? |  |
| If you suffer from incontinence, do you use any additional specialized treatments as pelvic floor muscle training (PFMT), physiotherapy or electrostimulation etc.?  If your answer is yes please specify. | Yes: 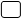  Specify:  No: 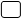 |
| Do you practice sports?  If your answer is yes, how often do you practice per week? | Yes: 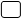  How often:  No: 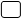 |
| Have you already practiced sexual intercourse after delivery?  If your answer is yes, how many weeks after delivery did you practice sexual intercourse? | Yes: 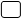  When:  No: 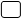 |
| Did you feel any changes during sexual intercourse after delivery?  If your answer is yes, please specify. | Yes: 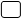  Specify:  No: 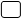 |
| Did you feel pain during sexual intercourse after delivery? | Yes: 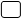  No: 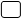 |
| Did you register foreign body feeling during sexual intercourse after delivery? | Yes: 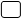  No: 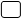 |
